# Assessment of Allergy to Milk, Egg, Cod, and Wheat in Swedish Schoolchildren: A Population Based Cohort Study

**DOI:** 10.1371/journal.pone.0131804

**Published:** 2015-07-02

**Authors:** Anna Winberg, Christina E West, Åsa Strinnholm, Lisbeth Nordström, Linnea Hedman, Eva Rönmark

**Affiliations:** 1 Department of Clinical Sciences, Pediatrics, Umeå University, Umeå, Sweden; 2 Department of Public Health and Clinical Medicine, Occupational and Environmental Medicine, the OLIN unit, Umeå University, Umeå, Sweden; Indiana University, UNITED STATES

## Abstract

**Objectives:**

Knowledge about the prevalence of allergies to foods in childhood and adolescence is incomplete. The purpose of this study was to investigate the prevalence of allergies to milk, egg, cod, and wheat using reported data, clinical examinations, and double-blind placebo-controlled food challenges, and to describe the phenotypes of reported food hypersensitivity in a cohort of Swedish schoolchildren.

**Methods:**

In a population-based cohort of 12-year-old children, the parents of 2612 (96% of invited) completed a questionnaire. Specific IgE antibodies to foods were analyzed in a random sample (n=695). Children reporting complete avoidance of milk, egg, cod, or wheat due to perceived hypersensitivity and without physician-diagnosed celiac disease were invited to undergo clinical examination that included specific IgE testing, a celiac screening test, and categorization into phenotypes of food hypersensitivity according to preset criteria. Children with possible food allergy were further evaluated with double-blind challenges.

**Results:**

In this cohort, the prevalence of reported food allergy to milk, egg, cod, or wheat was 4.8%. Food allergy was diagnosed in 1.4% of the children after clinical evaluation and in 0.6% following double-blind placebo-controlled food challenge. After clinical examination, children who completely avoided one or more essential foods due to perceived food hypersensitivity were categorized with the following phenotypes: allergy (29%), outgrown allergy (19%), lactose intolerance (40%), and unclear (12%).

**Conclusions:**

There was a high discrepancy in the prevalence of allergy to milk, egg, cod and wheat as assessed by reported data, clinical evaluation, and double-blind food challenges. Food hypersensitivity phenotyping according to preset criteria was helpful for identifying children with food allergy.

## Introduction

Reported food hypersensitivity (FHS) is common among children and adolescents [1, 2] and comprises different phenotypes. However, these phenotypes have not been thoroughly defined [3–6]. FHS is an umbrella term that includes reactions of both immunological origin (allergies) and non-immunological origin (intolerances) [7]. IgE-mediated allergies are more often severe enough to require emergency hospital care and are more frequently studied [1, 8] but other FHS phenotypes seem to be more common among schoolchildren with self-reported FHS [9]. Our knowledge about the phenotypes underlying reported FHS is still incomplete, partly due to the lack of studies [4, 5], and partly because the methods and diagnostic criteria used to identify children who are invited to undergo additional clinical evaluation are rarely described in detail [3, 6]. Independent of the underlying mechanisms perceived FHS often leads to elimination of essential foods [10] and may have a negative impact on the child’s quality of life [11]. Thorough clinical evaluation of a perceived FHS is therefore important.

Most population-based prevalence studies use questionnaire data, while some include IgE tests, and only a few validate the reported data using oral food challenges [1, 3, 4, 6]. Over-reporting of food allergy is common. Recent European meta-analyses showed a lifetime prevalence of reported food allergy of 17.9%, while the lifetime challenge-proven prevalence of allergy to eight common foods ranged from 0.1 to 0.6% [12, 13]. Skin prick testing (SPT) and measurements of specific IgE in serum have limitations, since IgE sensitization often occurs without symptoms [14, 15], and a substantial part of food allergies are non-IgE-mediated [1, 5, 16]. Therefore, food challenges are recommended for diagnosing food allergy, [17], and double-blind placebo-controlled food challenges (DBPCFC) are considered the gold standard [17, 18].

A previous study of this large, population-based cohort of 11–12-year-olds showed a reported prevalence of hypersensitivity to milk, egg, cod and/or wheat of 14.2% [2]. The primary aim of the current study was to investigate the prevalence of allergy to cow’s milk, hen’s egg, cod, and wheat according to reported data, clinical examination, and DBPCFC. A secondary aim was to describe the phenotypes of reported FHS in this cohort of Swedish schoolchildren.

## Materials and Methods

### Study population

**Fig 1:**
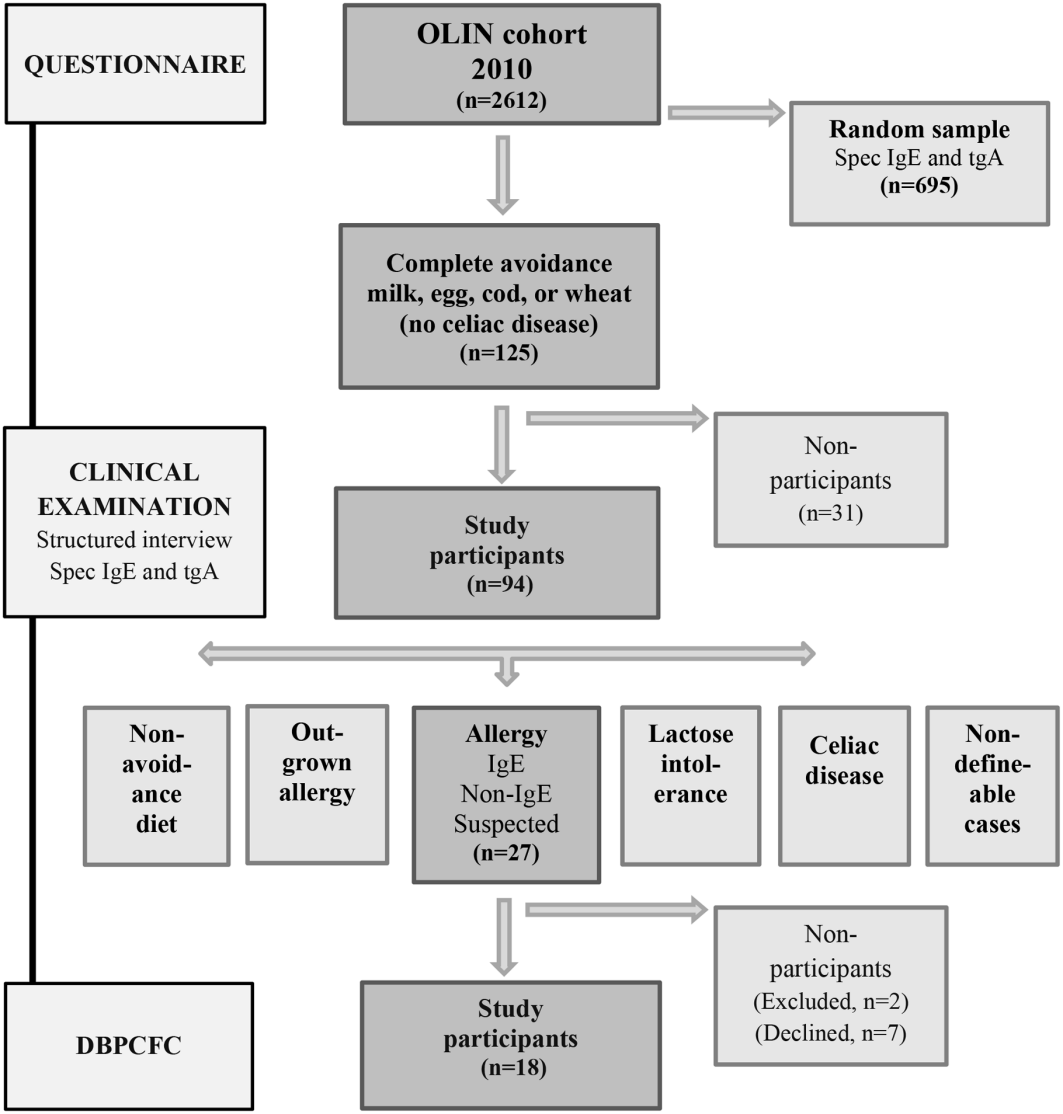
Study overview.

The pediatric cohort was established in 2006 as part of the Obstructive Lung Disease In Northern Sweden (OLIN) studies [19–21]. The parents of all children in first and second grade, aged 7–8 years, in three municipalities in Northern Sweden were invited to participate in a questionnaire study about asthma, rhinitis eczema and food hypersensitivity. Of these, the parents of 2585 children (96% of invited) completed the questionnaire. In 2010, there was a follow-up of the cohort where all children in fifth and sixth grade, now aged 11–12 years, in the same three municipalities were invited and the parents of 2612 children (96%) participated in the questionnaire [2,19]. A random sample from the cohort was also invited to donate blood to analyze the presence of IgE antibodies to foods and inhalant allergens and anti-tissue transglutaminase antibodies of IgA type (tTGA), and 695 (71%) children participated. This random sample was representative of the entire cohort [2]. Based on the results from the questionnaire sub-samples were invited to further examinations. A study overview is presented in Fig 1. Written informed consent was obtained from all children and from their legal guardians. The study was approved by the Regional Ethical Review Board in Umeå, Sweden.

### Questionnaire

The parental questionnaire included the International Study of Asthma and Allergies in Childhood (ISAAC) core questions [22] with additional questions about symptoms, physician diagnoses, medication, and possible determinants of asthma, rhinitis, and eczema. This questionnaire has been used in the OLIN-cohorts studies since 1996 [19, 20]. In 2006, questions regarding FHS questions were added [2, 21]. In the 2010 version of the questionnaire, the following questions were incorporated:

*To what extent does your child avoid the following foods; cow’s milk*, *hen’s egg*, *fish*, *or wheat*, *due to allergy/ hypersensitivity*? *(The alternatives for each food were; not at all/partially/completely)*

*Has your child been diagnosed by a physician as having celiac disease*?

The questionnaire also included questions about whether the child had ever needed emergency care because of a reaction to a food or if the child was prescribed epinephrine due to a food allergy. The questionnaires were distributed by school personnel.

### Clinical examinations

Children with reported FHS to cow’s milk, hen’s egg, fish, or wheat, complete avoidance of the culprit food and no physician diagnosed celiac disease were invited to undergo clinical evaluation. The clinical evaluation included a structured interview, physical examination, and blood tests to analyze specific IgE to the culprit food and tTGA. All clinical evaluations were performed by the same pediatric allergist (AW). The structured interview included FHS-related questions about age of onset; initial and current symptoms; symptom severity; time to symptom onset and symptom duration after intake of the culprit food; concomitant triggers; eliciting doses; dietary substitution products; allergic heredity; current eczema, rhinitis and/or asthma; and current physician-diagnosed celiac disease.

### Serum analyses

The serum samples were analyzed using a food screening test (the ImmunoCAP specific IgE blood test, fx5, ThermoFisher Diagnostics, Uppsala, Sweden) that included cow’s milk, hen’s egg, cod, soy, wheat, and peanut. If the screening test was positive, specific IgE was analyzed separately for all of the foods in the screening test. In serum samples from the children participating in the clinical examination, specific IgE to the culprit foods was analyzed. Inhalant allergen screening was also conducted (ImmunoCAP, ThermoFisher Diagnostics, Uppsala, Sweden) and included the following allergens: birch, timothy, mugwort, cat, dog, horse, dust mites (Dermatophagoides Pteronyssius and Farinae), and Claudosporium. An IgE serum level > 0.35 kU/L was considered positive for all of the IgE tests.

Further, tTGA was measured in serum as a marker of celiac disease using the EliA Celikey IgA test (ThermoFisher Diagnostics, and tTGA < 7 U/L was considered negative.

The celiac screening test was included in order to identify children with undiagnosed celiac disease.

### Criteria for FHS phenotypes

Based on the structured interview and on the analyses of specific IgE to the culprit foods and tTGA, the children were categorized according to FHS phenotype (S1 Table) using criteria that were set according to the WAO position paper on the terminology of FHS [7] and the current knowledge of common clinical presentations of FHS phenotypes in childhood and adolescence [23, 24, 25]. All mandatory criteria had to be fulfilled for the diagnosis of each FHS phenotype. For a diagnosis of food allergy, at least two secondary criteria also had to be fulfilled. If a child reported FHS symptoms to more than one food and the adverse reactions to the foods were categorized as different symptom phenotypes the child was sorted in the FHS symptom phenotype category with the highest priority. The priority order was set; IgE-mediated allergy > non-IgE-mediated allergy > suspected allergy > outgrown allergy and lactose intolerance > non-definable case. If a child was identified with undiagnosed celiac disease, the child was referred to a Pediatric clinic for further evaluation.

### Comparison of baseline characteristics between different FHS symptom phenotypes and a random population sample

In order to describe the children sorted into each FHS symptom phenotype, the different FHS groups; food allergy (IgE-mediated, Non IgE-mediated and suspected food allergy), outgrown food allergy and lactose intolerance were compared to the random population sample (n = 695) for the prevalence of the variables; male sex, positive IgE food screen, positive IgE inhalant screen, physician diagnosed asthma, rhinitis and eczema, living area, living in house or apartment, mother or father smoked and having older siblings.

### DBPCFCs

The inclusion criteria for invitation to undergo further evaluation with DBPCFCs were fulfillment of the criteria for allergy or suspected allergy to cow’s milk, hen’s egg, cod, and/or wheat. The exclusion criteria were anaphylactic reaction to the culprit food within the last 2 years or concomitant chronic disease that could aggravate the performance or interpretation of the DBPCFC. There were three challenge sessions for each food in random order, two with active substance and one with placebo. The challenge series was evaluated at a follow-up visit one week after the last challenge session. A DBPCFC was considered positive if the triggered symptoms were objective and/or reproducible or if the challenge triggered symptoms that were severe enough for the challenge series to be terminated. The DBPCFC method is described in detail in S1 file.

### Definitions

*Reported food allergy*—was defined as reported allergy/hypersensitivity to and complete avoidance of one or more of the foods; cow’s milk, hen’s egg, fish, or wheat and no physician diagnosed celiac disease (based on the questionnaire).

*Clinically evaluated food allergy*—included the FHS symptom phenotypes; IgE-mediated allergy, non IgE-mediated allergy and suspected allergy (based on structured interviews and specific IgE).

*Challenge proven food allergy*–was defined as a DBPCFC-series with a positive outcome

### Statistical analyses

Data were analyzed using IBM SPSS Software version 21 (New York, USA). The Chi-square test was used for comparison of proportions. A p-value <0.05 was considered statistically significant. Confidence intervals for proportions were calculated using the method of Agresti and Coull [26].

## Results

### Study participants

Of the entire cohort of 2612 children, 4.8% (95% CI 4–6%) (n = 125) had a reported allergy to of one or more of the foods; cow’s milk, hen’s egg, cod, or wheat. These children were invited to undergo a clinical examination, and 94 children (75%) participated. The demographic and clinical characteristics were similar among participants and non-participants in the clinical study (S2 Table).

### FHS phenotypes

The results of the FHS phenotyping are presented in Table 1. Of the 94 children who participated in the clinical examination, 87 (93%) reported symptoms to milk, 12 (13%) reported symptoms to egg, 16 (17%) reported symptoms to cod and 4 (4%) reported symptoms to wheat.

**Table 1:** Distribution of phenotypes of food hypersensitivity (FHS) based on the clinical examinations, and the relation to the individual triggering foods.

	Participants(n = 94)	Triggering foods *			
		Milk	Egg	Cod	Wheat
		(n = 87)	(n = 12)	(n = 16)	(n = 4)
FHS PHENOTYPE	n (%)	n (%)	n (%)	n (%)	n (%)
**ALLERGY**					
IgE-mediated allergy	18 (19)	3 (3)	8 (67)	12 (75)	1 (25)
Non-IgE-mediated allergy	6 (6)	6 (7)	0	0	0
Suspected allergy	3 (3)	0	2 (17)	3 (19)	0
Outgrown allergy	18 (19)	28 (32)	0	0	1 (25)
**LACTOSE INTOLERANCE**					
Lactose intolerance	27 (29)	29 (33)	0	0	0
Suspected lactose intolerance	11 (12)	11 (13)	0	0	0
**CELIAC DISEASE**	1 (1)	0	0	0	1 (25)
**NON-DEFINABLE CASES**					
Symptoms not definable	2 (3)	3 (3)	0	0	1 (25)
No blood analyses (specific IgE/tTGA)	7 (7)	6 (7)	2 (16)	1 (6)	0
**NON-AVOIDANCE DIET**	1 (1)	1 (1)	0	0	0

* Some of the children reported symptoms to more than one food.

#### Food allergy

A total of 18 children (19%) were diagnosed as having an IgE-mediated allergy to cow’s milk, hen’s egg, cod, or wheat. Six children (6%) had a non-IgE mediated allergy to one or more of these foods. In addition, 3 children with a convincing clinical history of a current food allergy but who lacked one criterion for a food allergy diagnosis were categorized as suspected allergy (Table 1). Based on the diagnostic phenotyping and assuming that the distribution of the FHS phenotypes were similar among the non-participants, the estimated prevalence of allergy to milk, egg, cod and wheat in the entire cohort of 2612 children was 1.4% (95% CI 1–2%)

An additional 18 children (19.1%) were diagnosed as having an outgrown food allergy phenotype (Table 1). These children had a convincing clinical history of previous allergy to cow’s milk, hen’s egg, cod, or wheat but could now tolerate at least 100 ml of milk and/or up to a portion size of egg, cod, or wheat without symptoms.

#### Lactose intolerance

Of the 94 children that underwent clinical examinations, 27 (29%) were diagnosed as having a lactose intolerance phenotype. In addition, 11 children (11.7%) had a history of suspected lactose intolerance but lacked one diagnostic criterion (Table 1).

#### Celiac disease

A physician diagnosis of celiac disease, reported by 8 out of 2612 children in the entire cohort, was an exclusion criterion for invitation to the clinical examination. Nonetheless, one of the children who underwent a clinical examination had a diagnosis of celiac disease. In this case, the physician’s diagnosis was made after the questionnaire study but prior to the clinical examination. None of the 94 children who participated in the clinical study had a positive celiac screening test (tTGA). Among the children in the randomized population sample, 7 of 695 (1%) had a positive tTGA.

#### Non-definable cases and the non-avoidance diet

In some cases, the symptoms and findings from the clinical evaluations did not fulfill the criteria for any of the pre-set phenotype categories. These cases were thus categorized as non-definable. This group included children who reported symptoms that were non-specific and children that declined blood sampling. One of the 94 children was no longer on an elimination diet (Table 1).

### Comparison of baseline characteristics between different FHS symptom phenotypes and a random population sample

Among children with a food allergy, the majority had a positive food (63%) or inhalant (74%) allergen screen test. Reported physician diagnoses of asthma (52%), rhinitis (44%), and eczema (67%) were very common in these children. The prevalence of positive food and inhalant screen tests, as well as the prevalence of physician diagnoses of asthma, rhinitis, and eczema among the children with an outgrown food allergy and a lactose intolerance phenotype was similar to the prevalence in a randomized population sample. (Table 2).

**Table 2:** Prevalence of the indicated variables in children according to their food hypersensitivity phenotypes compared to the prevalence in a random sample of children from the entire cohort.

	Food allergy n = 27	p-value*	Outgrown Allergy n = 18	p-value*	Lactose intolerance n = 38	p-value*	Random sample n = 695
	% (95% CI)		% (95% CI)		% (95% CI)		% (95% CI)
Male sex	52 (34–69)	0.972	44 (24–65)	0.554	34 (20–49)	0.038	52 (48–55)
Inhalant IgE screen	74 (58–90)	<0.001	44 (24–65)	0.695	29 (15–43)	0.180	40 (36–43)
Food IgE screen	63 (46–80)	<0.001	6 (n.a**)	0.195	11 (0–21)	0.289	17 (14–20)
Physician-diagnosed asthma	52 (34–69)	<0.001	17 (n.a**)	0.658	11 (0–21)	0.646	13 (11–16)
Physician-diagnosed rhinitis	44 (27–62)	<0.001	28 (8–47)	0.044	18 (6–31)	0.236	12 (10–14)
Physician-diagnosed eczema	67 (50–84)	<0.001	22 (4–41)	0.560	18 (6–31)	0.818	17 (14–20)

* Comparison between a random sample of the entire cohort and children with food allergy, outgrown food allergy, and lactose intolerance

** n.a: Sample prevalence too low for reliable calculation of confidence interval

There were no significant differences (p<0.05) between any of the different FHS phenotypes and the random population sample for the prevalence of the variables; living area, living in house or apartment, mother or father smoked or having older siblings.

### DBPCFC

Twenty-seven children with a food allergy were considered for further validation of their diagnosis using DBPCFC. Two cases were excluded from the invitation, one because of recent anaphylaxis caused by the culprit food and the other due to a concomitant chronic disease. Of the 25 invited children, 18 (72%) participated in 20 challenge series. Seven children declined participation; all of these children reported cod as the culprit food. Among the non-participants, the specific IgE to cod varied between 0.0–2.9 kU/L.

Of the 20 DBPCFC series, 9 were considered positive: 4 were performed with cow’s milk, 4 with hen’s egg, and 1 with cod. All but one child with a positive challenge outcome had a positive specific IgE test to the culprit food, and the level of specific IgE varied between 0.51–13.1 kU/L. Symptoms during the positive challenges were considered mild in 5 cases. These 5 children reacted with symptoms including stomach ache, diarrhea, eczema, itching of the mouth or mild swelling of lips. Of these, 2 children had late onset symptoms, which made a second active challenge necessary to prove that the symptoms were elicited by the challenge food. Four children reacted with anaphylaxis where after the challenge series was terminated. Among the children with anaphylaxis, 3 of 4 were challenged with hen’s egg and the specific IgE to the culprit food varied between 3.27–13.1 kU/L (Table 3). The cumulative challenge dose of hen’s egg protein that triggered severe reactions varied between 0.2–0.5 g. The study participant who had a severe reaction to cow’s milk experienced mild symptoms during the first active challenge with a cumulative dose of cow’s milk protein of 1.1 g. However, during the second active challenge, a cumulative dose of 0.04 g of milk protein triggered anaphylaxis. Assuming a similar distribution of a positive challenge outcome among the non-participants, the estimated prevalence of DBPCFC-proven food allergy to the essential foods (milk, egg, and cod) in the entire cohort would be 0.6% (95% CI 0–1%).

**Table 3:** Characteristics and challenge outcomes of the children (n = 18) who participated in the double-blind placebo-controlled food challenges series.

Sex	Challenge food	S-IgE Challenge food (kU/L)	Food IgE screening (kU/L)	Challenge outcome
**M**	Egg	0.52	0.91	0
**M**	Milk	0.00	0.04	0
**F**	Cod	8.03	7.37	mild
**F**	Cod	0.00	0.14	0
**F**	Milk	0.00	0.03	mild
**M**	Cod	0.00	0.40	0
**M**	Cod	0.00	0.11	0
**M**	Milk	3.27	36.10	severe
**M**	Milk	0.00	0.04	0
**F**	Milk	2.45	33.60	mild
	Egg	3.94	33.60	severe
**F**	Egg	0.80	1.11	mild
**F**	Milk	0.02	0.20	0
**M**	Egg	13.10	13.40	severe
	Cod	0.01	13.40	0
**F**	Milk	0.01	0.06	0
**F**	Egg	7.81	8.19	severe
**F**	Milk	0.08	0.20	0
**F**	Milk	0.51	2.96	mild
**F**	Egg	0.21	1.98	0

## Discussion

In this population-based Swedish cohort of 11–12-year old children, the prevalence of reported food allergy to cow’s milk, hen’s egg, cod, and/or wheat was 4.8%. The diagnosis of allergy to these foods after clinical evaluation was 1.4%, and the prevalence following DBPCFC was 0.6%. A previous study of the same cohort showed that perceived FHS was common; 26% of the children reported FHS to any food, and 14% reported FHS to one or more of the individual foods; milk, egg, cod or wheat [2]. Based on the results of the clinical evaluation the children in this study were categorized into FHS phenotypes according to preset criteria. This categorization gave a good overview of the distribution of phenotypes underlying perceived hypersensitivity to milk, egg, cod and wheat in this cohort and facilitated the identification of children with food allergy that were invited to undergo DBPCFC.

There was a high discrepancy in the prevalence of allergy to cow’s milk, hen’s egg, cod and/or wheat as estimated by reported data versus determination by clinical evaluation or DBPCFC. Notably, the prevalence of DBPCFC-proven allergy to milk, egg, cod, or wheat was 23-fold lower than the prevalence of reported FHS to these foods [2] and 8-fold lower than reported FHS to these foods and complete avoidance of the culprit food. Previous studies differ greatly in terms of the prevalence of challenge-proven food allergy found [6,13, 27], which at least partly can be explained by the use of different methods of evaluation [17]. In most studies, the identification of individuals from the population who were invited to undergo further evaluation of a food allergy was based on questionnaires and/or telephone interviews [3, 6]. To our knowledge, our study is the first to describe the identification of children with food allergy using FHS phenotyping based on predetermined criteria.

About one fourth of the children that underwent clinical examination were diagnosed as having a current allergy, either IgE- or non-IgE-mediated, to one or more of the individual foods; cow’s milk, hen’s egg, cod, or wheat. The majority of these had an IgE-mediated food allergy. In this category, the prevalence of positive food and inhalant allergen screening tests and physician diagnoses of asthma, rhinitis, and eczema were very high, which supports a valid diagnosis [15, 17, 28]. Half of the DBPCFCs were positive, and only one of the children with a positive challenge outcome was not sensitized to the culprit food. Four of nine children with a positive challenge had anaphylactic reactions. Only one of these had been prescribed adequate medications for their food allergy and concomitant asthma. These findings emphasize the importance of thorough evaluations of reported FHS to improve the management of children with food allergy [23].

Interestingly, 19% of the children reporting complete avoidance of milk, egg, cod, and/or wheat were diagnosed as having an outgrown food allergy phenotype. This finding is consistent with the natural course of allergy to foods like cow’s milk and hen’s egg, with a high rate of tolerance development [29]. However, although these children now tolerated fairly large amounts of the culprit foods, they were still on elimination diets either due to uncertainty about whether they had fully outgrown their allergy or because they disliked the taste or texture of the avoided food. These findings highlight the need for repeated evaluations of tolerance development and dietary restrictions in children with food allergy to prevent unnecessary food avoidance [23].

Among the children who underwent clinical examination, lactose intolerance was the most common FHS symptom phenotype. Even though a previous study showed a higher-than-expected prevalence of lactase down-regulation (14%) among Swedish school children [30], others have shown that over-reporting of lactose intolerance is frequent even in populations in which lactase down-regulation is common [31]. The true prevalence of lactose intolerance in our pediatric cohort could not be estimated from this study, since children with partial avoidance of milk were not invited to participate and children categorized as a lactose intolerance phenotype were not further evaluated.

Even though previous studies have shown a high prevalence of celiac disease among people with symptoms of lactose intolerance [32], only one child in our study had celiac disease, and none of the children who underwent clinical examination had a positive celiac screening test. Among the children in the random population sample who donated blood for celiac screening tests, the prevalence of a positive tTGA test was 1%. In addition, the reported prevalence of a physician diagnosis of celiac disease among the 2612 children in the cohort was 0.3%. Taken together, this means that the likely prevalence of diagnosed and undiagnosed celiac disease in the entire cohort is somewhat lower compared to other recent studies conducted in this region [33].

A major strength of our study is its population-based design and the high participation rates. The participants who underwent clinical examinations were representative of the invited participants. Also, suspected allergy to milk, egg, cod and wheat was validated according to the gold standard [17] using DBPCFC series and validated challenge recipes [34]. Nonetheless, even DBPCFCs have some limitations that could result in an under-diagnosis of food allergy. This includes the difficulty of hiding large enough doses of challenge foods in a suitable volume of vehicle [35]. In addition, a few children with food allergies require repeated exposure or concomitant triggers, such as exercise, for symptoms to be elicited [36]. To reduce the risk of missing food-allergic children who require repeated exposure to the culprit food, the challenge series in our study included two challenges with active substance. None of the participating children had a clinical history of exercise-induced anaphylaxis.

Since most children with allergies to foods like milk, egg and wheat develop tolerance during childhood [3, 28], there were relatively few children who were finally diagnosed as having a food allergy, even though they were selected from a large population-based cohort with a high prevalence of reported FHS [2]. Therefore the inclusion of cow’s milk, hen’s egg, cod and wheat only could be considered a study limitation. However, this study focused on these individual foods due to the high reported prevalence of FHS to milk, egg, cod and wheat in this cohort [2, 21,], the possible negative nutritional effects of elimination of these foods [37] and the lack of knowledge about the phenotypes underlying perceived FHS to milk, egg, cod, and/or wheat in schoolchildren [4, 5]. Further, our study focused mainly on the validation of food allergies. However, the majority of children in this cohort who reported perceived FHS to milk, egg, cod and/or wheat were not diagnosed as having a current food allergy. In order to fully understand the phenotypes underlying perceived FHS, all children with reported adverse symptoms to food would need to undergo further evaluation, including genotype testing for lactase non-persistence, lactose challenges, and blinded food challenges with adequate challenge doses of the culprit food [17].

## Conclusions

In this population-based cohort of Swedish 11–12-year-old school children, the prevalence of reported allergy to cow’s milk, hen’s egg, cod, or wheat was 4.8%, whereas the prevalence according to preset diagnostic criteria was 1.4%. This figure was further halved when possible food allergy was evaluated with DBPCFC. Based on the clinical examinations, the specific IgE analyses, and a celiac screening test, children with complete avoidance of one or more of the individual foods; milk, egg, cod or wheat due to perceived FHS were categorized into the following FHS phenotypes: allergy (29%), outgrown allergy (19%) or lactose intolerance (40%), or unclear (12%). In the future, FHS phenotyping according to preset criteria could be a helpful tool for describing the process of identifying of children that should be evaluated further for perceived FHS and for the choice of a suitable method for this assessment.

## Supporting Information

S1 File Double-blind placebo-controlled food challenges (DBPCFCs). (DOCX)

S1 Table Criteria for food hypersensitivity (FHS) phenotypes. (DOCX)

S2 Table Comparison of study participants and non-participants according to the indicated variables among the 125 children with food hypersensitivity who were invited to undergo clinical examination. (DOCX)
