# Supplementary material for: Assessment of Allergy to Milk, Egg, Cod, and Wheat in Swedish Schoolchildren: A Population Based Cohort Study
Source: PLoS One. 2015 Jul 2;10(7):e0131804. doi: 10.1371/journal.pone.0131804 (PMC4489866; doi:10.1371/journal.pone.0131804)
Supplement: S1 File — (DOCX) [file pone.0131804.s001.docx]

**S1. File**

**Double-blind placebo-controlled food challenges (DBPCFCs)**

The challenge series included three challenge occasions for each food, two with active substance and one with placebo, using previously validated recipes (24). The challenge sessions were set one week apart, and the serving order was drawn by lot. Both the patients and the study personnel conducting the challenges were blinded to the challenge food. The challenge doses for each culprit food, the total dose, and the specific and cumulative doses for each dose step are presented in Table 1:1. The challenge was started on step 1, 2, or 3 depending on the severity of previous reactions. The doses were given 30 minutes apart until objective symptoms were elicited or the final challenge dose was completed. An intravenous line was inserted prior to the challenge. Peak expiratory flow (PEF) and blood pressure were measured before and after the challenge and when symptoms occurred if applicable. The children were observed for 2 hours after each completed challenge and, after hospital departure, they completed a 3-day symptom diary. If a child had an anaphylactic reaction during the challenge, the challenge series was terminated. In case of anaphylaxis, the child was observed at the hospital for 4–12 hours after treatment. The length of observation depended on the severity of the reaction according to national guidelines [1].

The DBPCFs were performed at hospitals with a pediatric ward and with adequate emergency equipment that was immediately available. Challenges were not performed if the child had symptoms of an ongoing infection, allergy, or asthma. Oral antihistamines were not allowed three days prior to the food challenge, and oral corticosteroids were not allowed a week prior to the challenge session. The same experienced pediatric allergist and study nurse were present and responsible at all challenge occasions. The code was broken and the challenge series was evaluated at a follow-up visit one week after the last food challenge. A DBPCFC was considered positive if the triggered symptoms were objective and/or reproducible or if the challenge triggered symptoms that were severe enough for the challenge series to be terminated.

**S1 File Table 1**. Challenge doses, total dose, and specific and cumulative doses for each dose step for each culprit food.

| **Dose step** | **Test substance** | **Hen’s egg** (raw egg white) **protein dose** | | **Cow’s milk (**low fat milk) **protein dose** | | **Cod** (cod puree) **protein dose** | |
| --- | --- | --- | --- | --- | --- | --- | --- |
|  | Volume | Dose | Cumulative dose | Dose | Cumulative dose | Dose | Cumulative dose |
|  | (ml) | (g) | (g) | (g) | (g) | (g) | (g) |
| **1.** | 0.1 | 0.0006 |  | 0,0006 |  | 0.0005 |  |
| **2.** | 1.0 | 0.006 | 0.006 | 0.006 | 0.006 | 0.005 | 0.0051 |
| **3.** | 5.0 | 0.032 | 0.038 | 0.030 | 0.036 | 0.026 | 0.031 |
| **4.** | 20.0 | 0.128 | 0.166 | 0.120 | 0.156 | 0.102 | 0.133 |
| **5.** | 50.0 | 0.320 | 0.486 | 0.300 | 0.456 | 0.255 | 0.388 |
| **6.** | 100.0 | 0.640 | 1.126 | 0.600 | 1.056 | 0.510 | 0.898 |
| **7.** | 200.0 | 1.280 | 2.406 | 1.200 | 2.256 | 1.020 | 1.918 |
| **Total** | 376.1 |  | 2.406 |  | 2.256 |  | 1.918 |

**Reference**

1. Swedish Association for Allergology (SFFA: National guidelines for treatment of anaphylaxis; *www.*sffa.nu/Webbsidor/Diverse/2014_SFFA_Anafylaxi.pdf
