# Supplementary material for: Assessment of Allergy to Milk, Egg, Cod, and Wheat in Swedish Schoolchildren: A Population Based Cohort Study
Source: PLoS One. 2015 Jul 2;10(7):e0131804. doi: 10.1371/journal.pone.0131804 (PMC4489866; doi:10.1371/journal.pone.0131804)
Supplement: S1 Table — (DOCX) [file pone.0131804.s002.docx]

**S1 Table 1**. Criteria for food hypersensitivity (FHS) phenotypes.

| **FHS PHENOTYPE** | **CRITERIA** |
| --- | --- |
| **IgE-mediated**  **allergy*** | **Mandatory criteria**   - A positive IgE-test, provided there was exposure to the food within the previous 2 years - Symptoms triggered by less than 100 ml of milk or a portion size of egg, cod, or wheat products - Onset before 5 years of age, provided that the food was introduced in the child’s diet before that age   **Secondary criteria**   - First symptom within 15 minutes of exposure - Symptoms in more than one organ system - Symptoms are triggered by trace amounts of the food - Symptoms are triggered by skin exposure - Symptoms are triggered by airborne exposure - Anaphylaxis / exercise induced anaphylaxis |
| **Non-IgE mediated**  **allergy*** | **Mandatory criteria**   - A negative IgE test - Symptoms are triggered by less than 100 ml of milk or a portion size of egg, cod, or wheat products - No celiac disease - Onset before 5 years of age, provided that the food was introduced in the child’s diet before that age   **Secondary criteria**   - First symptom >1 hour after exposure - Symptom in more than one organ system - Symptoms are triggered by trace amounts of the food |
| **Outgrown**  **allergy**** | **Mandatory criteria**   - A convincing clinical history of IgE- or non-IgE-mediated allergy, but the child can now tolerate at least 100 ml of milk or a portion size of egg, cod, or wheat products |
| **Lactose**  **intolerance**** | **Mandatory criteria**   - Onset >5 years of age - Symptoms limited to flatulence, stomach-ache, and/or diarrhea - Symptoms are triggered by more than 100 ml of milk - Symptom-free on a lactose-free/lactose-reduced diet. - No celiac disease |
| **Celiac disease**** | **Mandatory criteria**   - A doctor´s diagnosis of celiac disease and/or a positive tTGA test |
| **Non-definable** | - Avoids milk, egg, fish, and/or wheat, but does not fulfill the criteria for any of the diagnosis groups - No blood analyses (specific IgE or tTGA) |
| **Non-avoidance diet** | - No longer on an elimination diet |

*All mandatory and at least 2 secondary criteria had to be fulfilled for diagnosis

**All mandatory criteria had to be fulfilled for diagnosis
