# Supplementary material for: Assessment of Allergy to Milk, Egg, Cod, and Wheat in Swedish Schoolchildren: A Population Based Cohort Study
Source: PLoS One. 2015 Jul 2;10(7):e0131804. doi: 10.1371/journal.pone.0131804 (PMC4489866; doi:10.1371/journal.pone.0131804)
Supplement: S2 Table — (DOCX) [file pone.0131804.s003.docx]

**S2. Table 2. Comparison of study participants and non-participants according to the indicated variables among the 125 children with food hypersensitivity who were invited to undergo clinical examination.**

|  | Participants  (n=94) | Non-participants  (n=31) |  | p-value |
| --- | --- | --- | --- | --- |
|  | % (95% CI) | % (95% CI) |  |  |
| Culprit food |  |  |  |  |
| Cow’s milk | 83 (75-91) | 77 (63-92) |  | 0.593 |
| Hen’s egg | 10 (3-16) | 13 (0-25) |  | 0.735 |
| Cod | 16 (9-25) | 19 (5-33) |  | 0.782 |
| Physician diagnosis |  |  |  |  |
| Asthma | 25 (17-34) | 19 (5-33) |  | 0.331 |
| Rhinitis | 29 (20-38) | 23 (8-37) |  | 0.326 |
| Eczema | 33 (24-42) | 28 (14-44) |  | 0.368 |
| Municipality |  |  |  |  |
| Luleå | 48 (38-58) | 42 (26-58) |  | 0.573 |
| Kiruna | 27 (18-35) | 23 (8-37) |  |  |
| Piteå | 25 (17-34) | 35 (19-51) |  |  |
